# The association between research design and the perceived treatment effectiveness: a cross-sectional study

**DOI:** 10.3389/fmed.2023.1220999

**Published:** 2023-12-22

**Authors:** Nensi Bralić, Ivan Buljan

**Affiliations:** ^1^Department of Research in Biomedicine and Health, University of Split School of Medicine, Split, Croatia; ^2^Faculty of Humanities and Social Sciences, Department of Psychology, University of Split, Split, Croatia

**Keywords:** hierarchy of evidence, study design, researcher, healthcare worker, consumer, treatment effectiveness

## Abstract

**Objective:**

To evaluate the impact of research design on the perceived medical treatment effectiveness among researchers, healthcare workers (HCWs) and consumers in Croatia.

**Methods:**

A cross-sectional study was conducted from November 2021 to February 2022 using an online survey. The participants were researchers, HCWs and consumers from Croatia. The survey had six scenarios about the same medical treatment presented within different study designs and in random order. Participants were asked to assess on a scale from 1 to 10 if the descriptions presented a sufficient level of evidence to conclude that the treatment was effective.

**Results:**

For researchers (*n* = 97), as the number of participants and degree of variable control in the study design increased, the perceived level of sufficient evidence also increased significantly. Among consumers (*n* = 286) and HCWs (*n* = 201), no significant differences in scores were observed between the cross-sectional study, cohort study, RCT, and systematic review.

**Conclusion:**

There is a need to implement educational courses on basic research methodology in lower levels of education and as part of Continuing Medical Education for all stakeholders in the healthcare system. Trial registration: this study has been registered on the Open Science Framework prior to study commencement (https://osf.io/t7xmf).

## 1 Introduction

The hierarchy of evidence refers to the presentation of levels of quality of evidence that can be obtained using different study designs. It is usually presented as a “pyramid of evidence” where systematic reviews and meta-analyses are placed on the top and observational studies at the bottom of the pyramid. The hierarchy of evidence was first introduced by the Canadian Task Force on the Periodic Health Examination in 1979 (1). It is a helpful tool for understanding the levels of evidence between different study designs (2).

When discussing evidence about health, it is generally referred to as evidence gathered from medical studies written in the form of scientific papers and published in peer-reviewed scientific journals. However, misinterpretation of evidence is a common problem in today’s society (3). With the increased use of social media, health-related information and misinformation about any topic are becoming more available and accessible (4, 5). Evidence-based medicine (EBM) is a movement that appeared in 1991, intending to develop clinical guidelines based on factual evidence and focusing on critical appraisal of the available evidence (6). While medical students have access to education on research methodology during their studies, most people have still not heard of different aspects of research methodology as it is not taught in lower levels of education or other non-biomedical higher education levels. Even though there has been an increase in interest in EBM through the years, most EBM educational interventions are still in need of improvement (7, 8).

Several studies have been conducted concerning consumers’ and medical professionals’ understanding of health information. A systematic review including 111 articles on health literacy among consumers found that low health literacy was associated with poor health-related knowledge, increased hospitalizations, and the use of different healthcare services (9). Another study on the critical appraisal skills of family physicians in Canada showed that even though participants were primarily young physicians, only about 50% understood critical appraisal concepts and how to apply them (10).

However, little is known about how different individuals in the healthcare system comprehend and apply scientific evidence and how well they understand the hierarchy of evidence. It would be expected for researchers and healthcare workers (HCWs) to increase their certainty about the effectiveness of the therapy when higher-level study designs are presented compared to lower-level study designs, while consumers might have trouble distinguishing different levels of evidence from the study designs as such knowledge is not taught in lower levels of education.

## 2 Materials and methods

### 2.1 Aim

This study aimed to assess the effect of research design on perceived medical treatment effectiveness among Croatian researchers, HCWs, and consumers.

### 2.2 Study design and setting

A quantitative cross-sectional study was used with HCWs, researchers and consumers from the Republic of Croatia. The responses were collected online for 3 months (November 2021 to February 2022).

### 2.3 Participants

Researchers, HCWs and consumers aged 18 years or older were included in this study as they all contribute to the final decision on a treatment option for a particular patient. Researchers bring new knowledge and findings to the table. Their work contributes to the expansion of the evidence basis for medical practices and treatments. HCWs apply that knowledge to diagnose, manage illnesses and treat their patients. Consumers are patients who are final healthcare service recipients and active participants in the shared decision-making process.

#### 2.3.1 Sampling

Convenience sampling, as well as snowballing, was used for the sampling strategy.

#### 2.3.2 Eligibility criteria

- A healthcare worker was defined as a medical doctor (MD) or doctor of dental medicine (DMD), either active or retired.
- A researcher was eligible for inclusion if they had a PhD from the field of biomedicine and health or at least one scientific paper from the field of biomedicine and health published in the last year, and if they were part of the scientific and teaching staff at a faculty.
- Consumers were patients who did not have a medical or dental medicine degree or a PhD in biomedicine and health.

### 2.4 Outcomes

#### 2.4.1 Independent variables

We collected information about age, gender, level of education, and occupation.

#### 2.4.2 Primary outcome: evidence sufficiency rating

Perceived sufficiency of presented evidence for each group to decide that the treatment is effective, measured using a scale of 1 to 10 for each scenario, where “1” means there is no evidence at all that a treatment is effective, and “10” means there is enough evidence.

#### 2.4.3 Secondary outcome: treatment effectiveness assessment

An estimate of treatment effectiveness measured on a scale of 1–10, with “1” meaning the treatment was not effective and “10” meaning the treatment was completely effective.

### 2.5 Data collection

The data was collected via a questionnaire created on the SurveyMonkey® platform (Supplementary material S3).^1^ The questionnaire and the included scenarios were designed by study authors who are experienced medical and psychology educators and research methodologists. Scenarios were then reviewed by two independent experts, who assessed their face validity, clarity, and relevance and offered guidance for revision. The pooled knowledge of the team ensured that study designs were accurately represented. The questionnaire was distributed to participants using publicly available electronic mail addresses and social media posts. Remainders were sent two weeks to a month after the first contact.

### 2.6 Scenario description

Within an online questionnaire, a series of scenarios about a single treatment for a particular disease within different levels of evidence were presented in a randomly ordered sequence to the participants. A ‘Page randomization’ option from the ‘Design survey’ section on the SurveyMonkey® platform was used to randomize the order of scenarios for each participant. Each scenario described a fictional study about the effectiveness of a single treatment against a particular disease. For each scenario, a different level of evidence was presented (case report, case series, cross-sectional study, cohort study, randomized controlled trial and systematic review of the literature). Participants then had to decide for every scenario if that description presented enough evidence to conclude that the treatment was effective and to estimate how effective the treatment was on a scale of 1–10. Independent variables collected were age, gender, level of education, and occupation. This study was registered on the Open Science Framework before study commencement.^2^

### 2.7 Bias

An order bias could have occurred if the scenarios within the questionnaire were presented simultaneously or gradually in the order of level of evidence. Participants could then clearly identify the critical differences between scenarios and easily observe the increase in the level of evidence throughout the scenarios, influencing their responses. In order to minimize that bias, scenarios in the questionnaire were presented in a randomly ordered sequence to the participants.

### 2.8 Sample size calculation

We hypothesized that the expected difference in the perceived effectiveness of the treatment would be 1 point on a scale from 1 to 10, with a standard deviation of 2. Based on those parameters, with 80% power and a 5% alpha error rate, we calculated that we would need 63 participants per group to observe the hypothesized difference. We used an online sample size calculator.^3^

### 2.9 Statistical analyses

Only the complete responses were included in data analyses. Demographic data were presented as frequencies and percentages for categorical data. Data normality was assessed using the Shapiro–Wilk test. For the numerical values for the entire group, data were presented as median with the interquartile range since the data distribution was asymmetrical. Group scores for numerical variables were presented as medians with 95% confidence intervals. For testing differences between the three groups, we used the Kruskal-Wallis test. To test the differences in scores on decision assessment, we used the Friedman test. Analyses were performed using JASP software, version 0.16.1 (JASP Team, 2022) and MedCalc software, version 20.027 (MedCalc Software, Ostend, Belgium). The significance level was set at *p* < 0.05.

## 3 Results

Invitations were sent to a total of 1,816 publicly available electronic mail addresses owned by researchers and HCWs. An automatic reply saying the message was delivered came back from 1,437 (79.13%) addresses. The message was not delivered to 254 (13.99%) addresses, and an automatic reply about the delivery status was not received for 125 (6.88%) addresses. To reach the consumers, invitations were sent to the electronic addresses of the representatives of the 171 citizens’ associations in Croatia, asking them to disseminate the invitation with the link to their members. The automatic reply with the delivery status was received for all addresses, and the message was delivered to 156 (91.23%) addresses. Additionally, the link was disseminated through private profiles and pages on social media (Facebook, Meta Platforms, Inc.).

**Figure 1:**
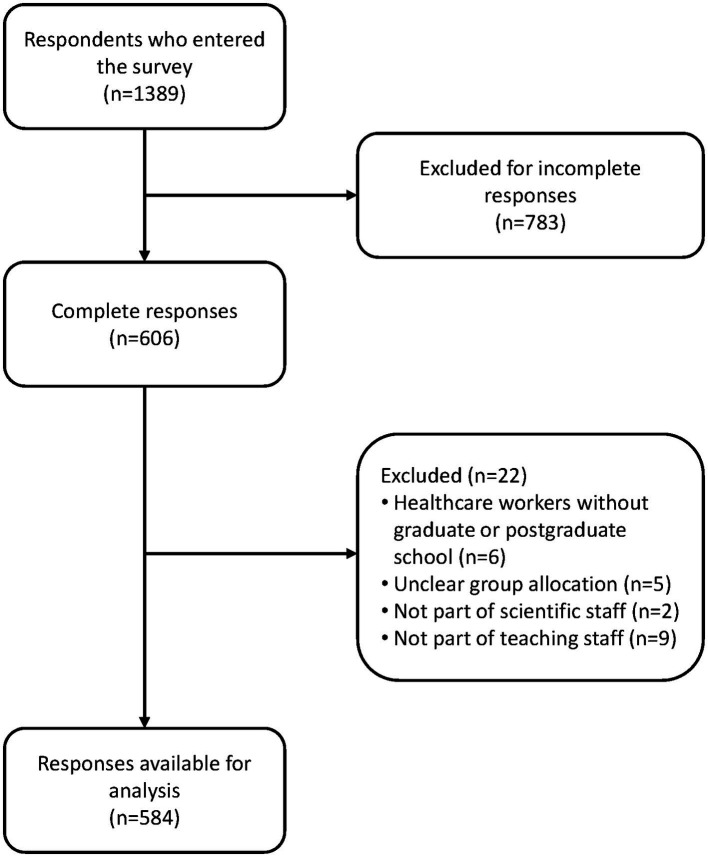
The flow of the participants.

A total of 1,389 respondents entered the link to the survey; 783 were excluded for not providing all relevant responses. An additional 22 responders were excluded for not meeting the eligibility criteria. Eleven researchers were excluded for not being part of a faculty’s scientific and teaching staff. In the end, 584 participants were eligible for analysis (97 researchers, 201 HCWs and 286 consumers) (Figure 1).

Participants were mainly women (74.3%), and the median age in years was 43.5 (IQR 33–52). Researchers and HCWs completed graduate or postgraduate schools, while the consumers mainly completed high school and graduate school (Table 1).

**Table 1:** Demographic characteristics of the participants.

**Variables**	**Total** **(*n* = 584)**	**Researchers (*n* = 97)**	**Healthcare workers (*n* = 201)**	**Consumers (*n* = 286)**	***p* value**
Women (*n*, %)	434 (74.3)	65 (67)	144 (71.6)	225 (78.7)	0.043^*^
Age (Md, IQR)	43.5 (33–52)	46 (38–53)	42 (31–51)	44 (34–52)	0.061^†^
Education (*n*, %)					
Primary school	3 (0.5)	0	0	3 (1)	<0.001*
Secondary school	113 (19.3)	0	0	113 (39.5)	
College	23 (3,9)	0	0	23 (8)	
Undergraduate school	22 (3.8)	0	0	22 (7.7)	
Graduate school	191 (32.7)	9 (9.3)	103 (51.2)	79 (27.6)	
Postgraduate school	208 (35.6)	88 (90.7)	98 (48.8)	22 (7.7)	
University student	24 (4.1)	0	0	24 (8.4)	

Md, median; IQR, interquartile range.

*Chi-square test.

^†^Kruskal-Wallis test.

### 3.1 Level of evidence needed to make an informed decision

Scores for the level of evidence needed to make an informed decision for all participants rose with the increase in the level of evidence, with statistically significant differences in scores between all study designs except for RCT and systematic review.

**Figure 2:**
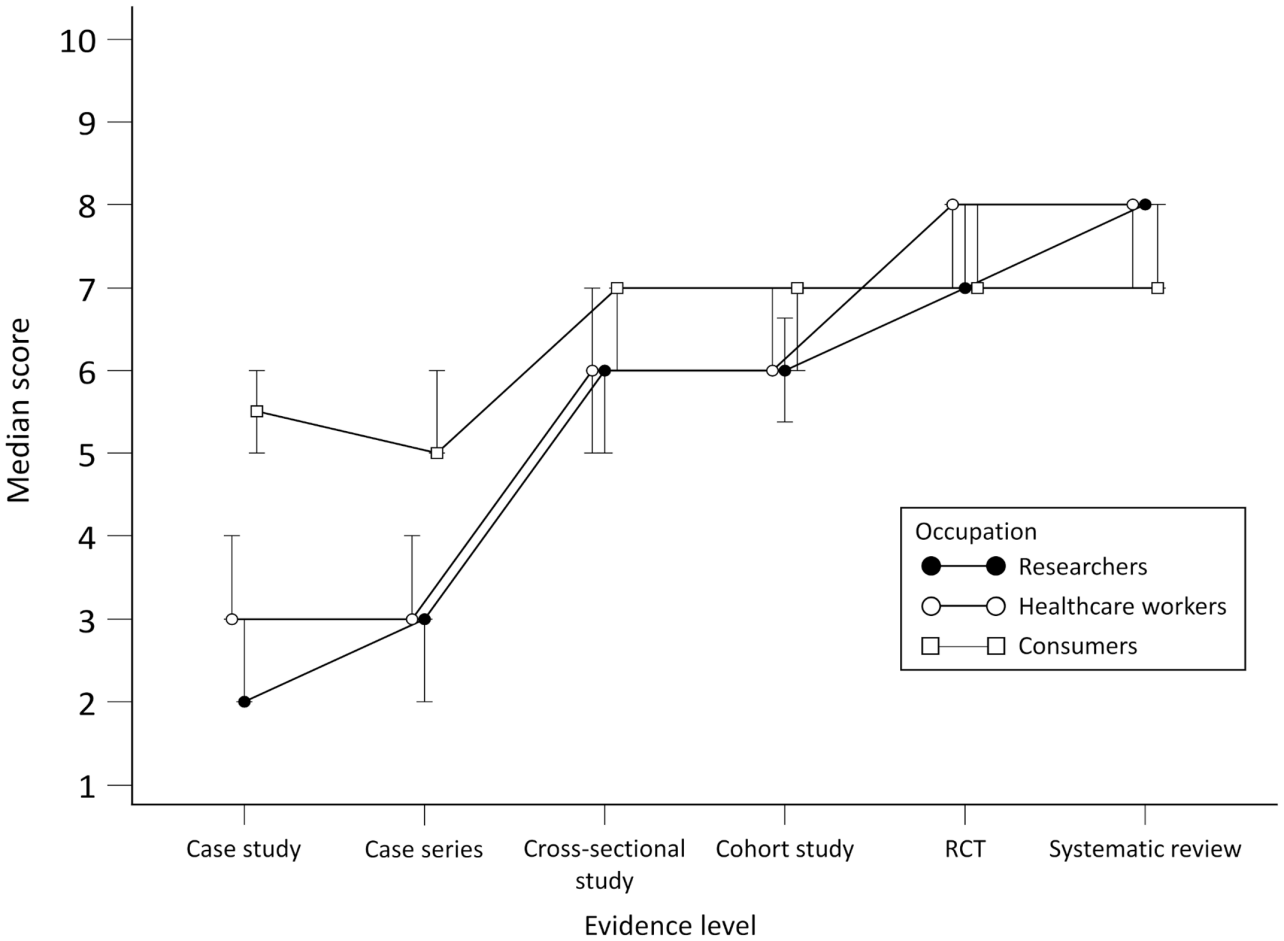
Scores per group for perceived adequacy of evidence about the effectiveness of the treatment.

No statistically significant differences for researchers were observed in scores between case study and case series and between RCT and systematic review (Figure 2). There were differences between researchers and consumers in scores for all study designs except RCT. When comparing researchers with HCWs, differences were found in case series and cross-sectional study scores.

There were no statistically significant differences for HCWs in scores between case study and case series, cross-sectional study and cohort study and between RCT and systematic review. There were differences in scores for the case study, case series, RCT, and systematic review for HCWs and consumers.

Consumers generally gave higher scores for the perceived adequacy of evidence about the effectiveness of the treatment for the case study and case series but lower for systematic reviews compared to researchers and HCWs. No statistically significant differences for consumers were observed in scores between the cross-sectional study and cohort study, cohort study and cross-sectional study and between RCT and systematic review. Scores per group throughout study designs are presented in Figure 2. Differences in scores for each scenario between the groups and comparison between scenario levels for each group are given in [see Supplementary material S1].

### 3.2 Treatment effectiveness assessment

Scores for all participants for the perceived efficacy of the treatment presented in different scenarios rose with the increase in the level of evidence, with statistically significant differences in scores between all study designs except for RCT and systematic review.

No statistically significant differences for researchers were observed in scores between case study and cross-sectional study, cohort study and RCT and between RCT and systematic review. There were differences between researchers and consumers in scores for all study designs except cohort study and RCT. When comparing researchers to HCWs, differences were found in case series and RCT scores.

For HCWs, there were statistically significant differences in scores between all study designs except RCT and systematic review. When compared to consumers, there were differences in scores for all study designs except the cohort study.

**Figure 3:**
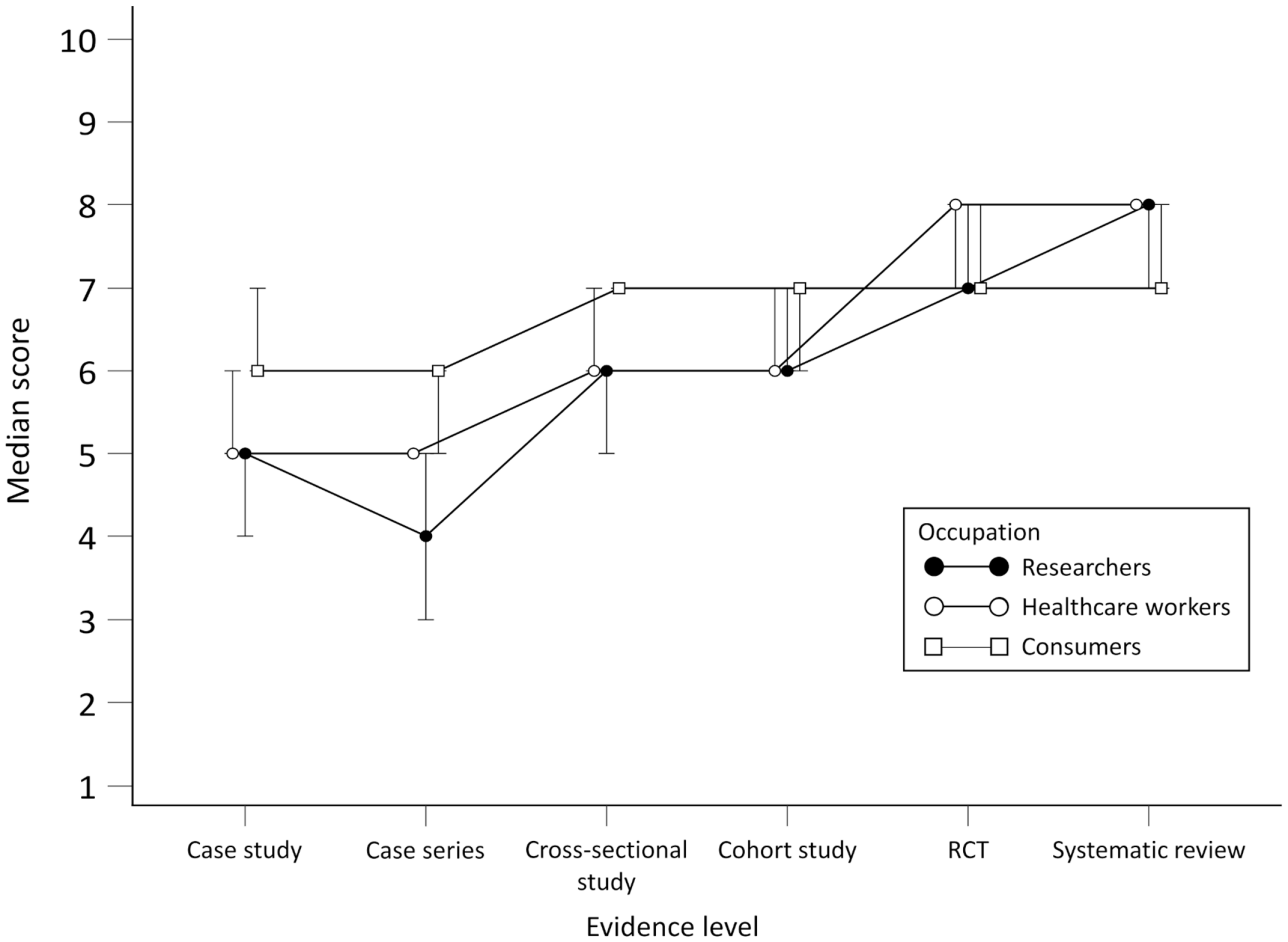
Scores per group for perceived efficacy of treatment.

Consumers generally gave higher scores for the perceived efficacy of treatment for the case study and case series but lower for systematic reviews compared to researchers and HCWs. No statistically significant differences for consumers were observed in scores between the case study and cross-sectional study, cross-sectional study and cohort study and between RCT and systematic review. Scores per group throughout study designs are presented in Figure 3. Differences in scores for each scenario between the groups and comparison between scenario levels for each group are given in [see Supplementary material S2].

## 4 Discussion

Our cross-sectional survey-based study showed significant differences in how different stakeholders in the healthcare system perceive and understand the evidence and different study designs. Differences in all outcomes were found between experts and consumers. Consumers were least capable of distinguishing between study designs as there were almost no differences in their perceived adequacy of evidence or treatment efficacy throughout scenarios. They gave the highest scores for the case study and the lowest for the systematic review compared to other groups.

This study tried to assess the concept of evidence in scientific research since that term became more emphasized in recent years, especially when facing the pandemic (11). Almost all media outlets were filled with health information, both correct and misinterpreted, and it was important to identify what was the evidence (12–14). This “infodemic”, as it was named by the Director General of the World Health Organization (WHO), made it much harder to discern between reliable and unreliable health information successfully, especially about the potential treatments, spreading and outcomes (15). The infodemic significantly impacted the spread of misinformation through social media, and people had trouble finding and understanding information about treatment, symptoms and prevention of the infection (16, 17). Therefore, the research on understanding the levels of evidence may be informative when sharing information with wider audiences. The results of the current study were in accordance with the study conducted on HCWs in Canada, which showed a general lack of critical appraisal skills among young physicians (10), but the novelty of our study is the inclusion of other populations in the research. As expected, researchers showed the best knowledge of the hierarchy of evidence, with the highest scores given for RCT and systematic review and the lowest for the case study and case series, compared to HCWs and consumers. Consumers were least capable of differentiating between study designs and gave similar scores for all study designs. HCWs from our sample had trouble differentiating between analytical observational studies and experimental and secondary study designs (based on experimental research), which may also have implications for physicians’ understanding of evidence. Future studies should investigate whether the understanding of the levels of evidence has implications in everyday physician practice.

Researchers and HCWs are both educated in research methodology during their studies and practice. In contrast, consumers had no formal education in such topics as research methodology is still not taught systematically in lower levels of education. However, as patient empowerment has become an important factor in healthcare decision-making nowadays, education about health and research in biomedicine and health for all patients has become more critical than ever. Furthermore, what is most concerning are HCWs’ scores for the level of evidence needed to make an informed decision and treatment effectiveness assessment. Since they are expected to provide the best treatment to their patients, they need to be able to find and critically appraise evidence pertaining to their field of expertise. As observational studies can not provide information on treatment effectiveness, it can not be acceptable for evidence acquired from a cross-sectional study to be perceived as adequate as evidence from an RCT or a systematic review. This study has pointed out the need to implement educational courses about basic research methodology in primary or secondary schools, as well as integrate compulsory courses on research in biomedicine and health as part of the Continuing Medical Education (CME) for HCWs.

This study was conducted on participants from Croatia, which should be considered when analyzing these results. The educational system in general and education on research methodology for different stakeholders in the healthcare system in Croatia can be different from education systems elsewhere. In addition, researchers, HCWs, and consumers included in our study might have different backgrounds and experiences, leading to varying understandings of study designs, which should be considered when generalizing the results of this study. Moreover, it was impossible to calculate our study’s overall response rate as we used several ways to disseminate the link to the questionnaire. The response rate could only be calculated for individuals who received the invitation via electronic mail directly. However, it was impossible to estimate the precise number of people exposed to the social media posts, the number of members of the citizens’ associations that received the forwarded invitations by their representatives or the number of people reached using the snowballing sampling strategy. Also, even though we tried to formulate the scenarios in the best way to avoid the possibility of response bias, some participants might have responded to the questions based on factors other than the content of the scenarios. A recommendation for future studies would be to conduct an even more rigorous validation of the scenarios for which methodological approaches already exist (18).

On the other hand, our study has several strengths. Scenarios in the questionnaire were presented in a randomly ordered sequence for each participant to prevent order bias, which could have occurred if the scenarios had been presented simultaneously or gradually. There was no mention of the exact study design for each scenario to prevent the use of external sources for determining the hierarchy of designs. Also, the questionnaire completion time was under 10 min to ensure the collection of high-quality responses from participants.

Future multi-national studies are needed to confirm these results and provide a deeper insight into the problem. Additionally, studies on the efficacy of research methodology educational programmes in lower levels of education and as part of the CME for different stakeholders in the healthcare system are needed.

## 5 Conclusion

When measuring the perceived adequacy of evidence and treatment effectiveness, researchers distinguished between different study designs better than HCWs and consumers. HCWs had trouble differentiating between any study design except case study and case series, and consumers were least capable of distinguishing between different study designs. There is a need to implement educational courses in lower levels of education and as part of the CME for all stakeholders in the healthcare system. Further research on the topic is needed to confirm these results.

## Data availability statement

The original contributions presented in the study are included in the article/Supplementary material, further inquiries can be directed to the corresponding author/s.

## Ethics statement

The studies involving humans were approved by the Ethics Committee of the University of Split School of Medicine. The studies were conducted in accordance with the local legislation and institutional requirements. The participants provided their written informed consent to participate in this study.

## Author contributions

NB contributed to data curation, formal analysis and interpretation of the data, writing the original draft, reviewing, and editing the final version of the manuscript. IB contributed to the conceptualization and design of the work, formal analysis, interpretation of the data, writing the original draft, and reviewing and editing the final version of the manuscript. All authors contributed to the article and approved the submitted version.

## Footnotes

^1^https://www.surveymonkey.co.uk/

^2^https://osf.io/t7xmf

^3^https://select-statistics.co.uk/calculators/sample-size-calculator-two-means/
