# Supplementary material for: The association between research design and the perceived treatment effectiveness: a cross-sectional study
Source: Front Med (Lausanne). 2023 Dec 22;10:1220999. doi: 10.3389/fmed.2023.1220999 (PMC10774223; doi:10.3389/fmed.2023.1220999)
Supplement: Supplementary file 1 [file Data_Sheet_1.DOCX]

**Supplementary material S1.** Differences for each scenario score between three groups and comparison between scenario levels for each group (perceived adequacy of the evidence).

| **Variables (Md, 95% CI)** | **Overall sample (n=584)** | **Researchers (n=97)** | **Healthcare workers (n=201)** | **Consumers (n=286)** | **P*** |
| --- | --- | --- | --- | --- | --- |
| Case study | 5 (4 to 5) | 2 (2 to 3)^#^ | 3 (3 to 4)^#^ | 5.5 (5 to 6)^‡^ | <0.001 |
| Case series | 4 (4 to 5) | 3 (2 to 3) | 3 (3 to 4) | 5 (5 to 6) | <0.001^§^ |
| Cross-sectional study | 6 (6 to 6) | 6 (5 to 6)^‡^ | 6 (5 to 7)^b^ | 7 (6 to 7)^b^ | 0.003 |
| Cohort study | 6 (6 to 7) | 6 (5,37 to 6,63) | 6 (6 to 7)^ǁ^ | 7 (6 to 7)^a^ | 0.151 |
| RCT | 7 (7 to 8) | 7 (7 to 8)^ǁ^ | 8 (7 to 8) | 7 (7 to 8) | 0.003 |
| Systematic review | 8 (7 to 8) | 8 (8 to 8)^a^ | 8 (7 to 8)^a^ | 7 (7 to 8)^‡a^ | 0.004 |
| P† | <0.001 | <0.001 | <0.001 | <0.001 |  |

Md median, CI confidence interval

^*^Kruskal-Wallis test.

^†^Friedman test.

^‡^Different from the other two groups.

^§^All groups are different

^ǁ^Not different from the other two groups

^#^Not different from the case series

^a^Not different from RCT

^b^Not different from the cohort study

**Supplementary material S2.** Differences for each scenario score between three groups and comparison between scenario levels for each group (perceived efficacy of the treatment).

| **Variables (Md, 95% CI)** | **Researchers (n=97)** | **Healthcare workers (n=201)** | **Consumers (n=274)** | **P*** |
| --- | --- | --- | --- | --- |
| Case study | 5 (4 to 5)^#^ | 5 (5 to 6) | 6 (6 to 7)^‡#^ | <0.001 |
| Case series | 4 (3 to 5) | 5 (5 to 5) | 5 (5 to 6) | <0.001^§^ |
| Cross-sectional study | 6 (5 to 6) | 6 (6 to 7) | 7 (7 to 7)^‡^ | <0.001 |
| Cohort study | 6 (6 to 7)^a^ | 7 (6 to 7) | 7 (6 to 7)^#^ | 0.296^ǁ^ |
| RCT | 7 (7 to 8) | 8 (7 to 8)^‡^ | 7 (7 to 8) | 0.018 |
| Systematic review | 8 (7 to 8)^a^ | 8 (8 to 8)^a^ | 7 (7 to 8)^‡a^ | 0.002 |
| P^†^ | <0.001 | <0.001 | <0.001 |  |

Md median, CI confidence interval

^*^Kruskal-Wallis test.

^†^Friedman test.

^‡^Different from the other two groups.

^§^All groups are different

^ǁ^No differences between groups

^#^Not different from a cross-sectional study

^a^Not different from an RCT

**Supplementary material S3.** English version of the questionnaire. The questionnaire was translated by Nensi Bralić and then back-translated to Croatian by an independent researcher who was not involved in the study.

**Scenarios about scientific research**

Dear Sir or Madam,

Before you is a survey used as a research instrument for the project " Professionalism in Health: Decision Making in Practice and Research – ProDeM" financed by the Croatian Science Foundation (IP-2019-04-4882), which is carried out at the Faculty of Medicine of the University in Split. In this study, we want to find out how you evaluate information about health and the ways in which you evaluate the effectiveness of health therapies.

Your answers are completely anonymous, they will be used only for research purposes and analyzed on a group level, and only researchers will have access to your data.

The survey is voluntary and intended for all adult citizens of the Republic of Croatia.

If you do not want to participate in the research, you are free to stop filling out the questionnaire at any time, and your data will not be taken into account during the analysis if you do not wish to do so. The study was approved by the Ethics Committee of the University of Split School of Medicine, Class 003-08/21-03/0003 No. 2181-198-03-04-21-0084.

For further information about the research, you are free to contact the research manager Nensi Ćaćić via email: [nensi.cacic@mefst.hr](mailto:nensi.cacic@mefst.hr).

Please read the offered scenarios and questions and answer them by choosing the offered answers in the provided place. We sincerely appreciate your feedback and thank you for your patience and affability.

1. I agree to participate in this research.

- Yes
- No

2. How old are you? *(please state only the number)*

_________________

3. Gender:

- Male
- Female

4. Completed level of education:

- Primary school
- Secondary school
- College
- Undergraduate school
- Graduate school
- Postgraduate school
- Currently a university student

5. Occupation *(choose the one in which you spend* ***the most time****)*

- Healthcare worker (MD or DMD) – currently employed, unemployed or retired
- Researcher – had a PhD from the field of **Biomedicine and Health** in the last two years **OR** at least one scientific paper from the field of **Biomedicine and Health** published in the last year
- I do not have an occupation, I am currently **studying**
- Other (please state): _____________________________________

6. Position within the faculty: **only for researchers*

- Scientific and teaching staff
- Teaching staff
- Scientific staff

7. Do you have a PhD?

- Yes
- No

8. Have you published a scientific paper in the last year?

- Yes
- No

9. What year did you get your doctorate? *(please state only the number of the year)*

____________________

On the following pages, you will be shown 6 scenarios of scientific research. Please read them carefully and answer the two questions below each scenario.

**Scenario**

This research aimed to evaluate the effectiveness of Drug X in relieving pain within a week after tooth extraction.

A participant is a 45-year-old male who came to the dental office complaining of pain in the lower jaw area on the right. In the medical history, he states that his lower first molar on that side has occasionally had spontaneous pain for the past month and that the day before he ate cherries and bit the pit with that tooth.

Examination of the mouth revealed a deep caries on the lower right first molar, and it was evident that the tooth had cracked from the crown to the root. An X-ray of the tooth showed inflammation under the root of that tooth and a tooth fracture that reached the bottom of the root. After that, the tooth was extracted, the alveolus was cleaned, and the patient was sent home.

As the occurrence of pain was expected for the next few days, the patient was recommended to drink painkiller Drug X twice a day (every 12 hours) for seven days and was asked to record his pain level on a scale of 1 to 10 four times a day (1 hour before taking Drug X and 4 hours after).

After a week, the patient returned to the office for a check-up. The subject states that the pain significantly decreased within 4 hours after taking medicine and does not mention side effects related to taking the therapy.

10. In your opinion, what level of evidence does this scenario provide for the effectiveness of Drug X?

| (it provides no evidence)  1 | 2 | 3 | 4 | 5 | 6 | 7 | 8 | 9 | (it provides all the evidence)  10 |
| --- | --- | --- | --- | --- | --- | --- | --- | --- | --- |
| ° | ° | ° | ° | ° | ° | ° | ° | ° | ° |

11. How effective is Drug X?

| (not at all effective)  1 | 2 | 3 | 4 | 5 | 6 | 7 | 8 | 9 | (it is completely effective)  10 |
| --- | --- | --- | --- | --- | --- | --- | --- | --- | --- |
| ° | ° | ° | ° | ° | ° | ° | ° | ° | ° |

**Scenario**

This research aimed to evaluate the effectiveness of Drug X in alleviating pain in the temporomandibular (chewing) joint area.

Four participants (two men and two women) participated in the research. All participants had pain symptoms in the temporomandibular (chewing) joint that occurs in the morning. The female participants were 43 and 29 years old, and the male participants were 64 and 32 years old. One female participant suffered from and was taking therapy for epilepsy, and the other had given birth 6 months prior and was currently breastfeeding. One male participant wore total dentures, and the other was infected with the hepatitis B virus. All the respondents were absent from work due to pain in the last month and felt pain a certain number of other days in the month. The participants were prescribed preventive medication of painkiller Drug X in the evening before going to bed for a month and were instructed to monitor the number of days they felt joint pain when waking up and the number of days they were absent from work.

After a month, the participants returned for a check-up. The first participant stated that she missed significantly fewer days from work than the month before and felt somewhat less pain during the past month. The second participant stated that during the past month, he did not miss work due to pain and felt significantly less pain than in the previous month. The third participant stated that during the last month, he missed somewhat fewer days from work and felt somewhat less pain than in the previous month. The fourth participant stated that she missed significantly fewer days from work during the last month and felt somewhat less pain.

The participants did not mention side effects related to taking the therapy

12. In your opinion, what level of evidence does this scenario provide for the effectiveness of Drug X?

| (it provides no evidence)  1 | 2 | 3 | 4 | 5 | 6 | 7 | 8 | 9 | (it provides all the evidence)  10 |
| --- | --- | --- | --- | --- | --- | --- | --- | --- | --- |
| ° | ° | ° | ° | ° | ° | ° | ° | ° | ° |

13. How effective is Drug X?

| (not at all effective)  1 | 2 | 3 | 4 | 5 | 6 | 7 | 8 | 9 | (it is completely effective)  10 |
| --- | --- | --- | --- | --- | --- | --- | --- | --- | --- |
| ° | ° | ° | ° | ° | ° | ° | ° | ° | ° |

**Scenario**

In this study, the authors wanted to evaluate the effectiveness of painkiller Drug X in adult patients suffering from periodontitis Stage 3, Grade B, who rated their pain as 4 or more on a scale of 1 to 10, and who were taking painkiller Drug X as directed by periodontology specialists regularly in the last year.

Twenty-five periodontology specialists from the Republic of Croatia distributed the survey to their patients (who meet the criteria for inclusion in this study) when coming for a pre-arranged check-up. The survey consisted of questions related to health-related quality of life and pain assessment after using the assessed drug.

Finally, 267 participants completed the survey (125 men and 142 women).

The results show that 75% of people felt that Drug X helped.

14. In your opinion, what level of evidence does this scenario provide for the effectiveness of Drug X?

| (it provides no evidence)  1 | 2 | 3 | 4 | 5 | 6 | 7 | 8 | 9 | (it provides all the evidence)  10 |
| --- | --- | --- | --- | --- | --- | --- | --- | --- | --- |
| ° | ° | ° | ° | ° | ° | ° | ° | ° | ° |

15. How effective is Drug X?

| (not at all effective)  1 | 2 | 3 | 4 | 5 | 6 | 7 | 8 | 9 | (it is completely effective)  10 |
| --- | --- | --- | --- | --- | --- | --- | --- | --- | --- |
| ° | ° | ° | ° | ° | ° | ° | ° | ° | ° |

**Scenario**

The purpose of this study was to assess the effectiveness of Drug X in relieving pain.

Participants in this study were students of the University of Split School of Medicine (320 respondents, 195 women and 125 men) who stated that they had a need to take painkillers at least once a month.

Students were divided into two groups (160 respondents per group). The participants themselves chose which drug and what quantity they wanted to take. When they felt the need to take a painkiller, participants in one group took Drug X (intervention group), and participants in the other group took the drug they usually take when they need a painkiller (control group).

The participants were followed for three years, and at the end, they filled out a survey in which they rated their overall satisfaction with the medicine they were taking, the effectiveness of the medicine, the duration of effect of the medicine, and listed all short-term and long-term side effects after taking medicine on a scale of 1 to 10.

Compared to the control group, participants in the intervention group were generally more satisfied with the treatment. The mean score on a scale of 1 to 10 for the effectiveness of the drug was higher in the intervention group than in the control group. Participants in the intervention group reported a more prolonged effect of the drug and, compared to participants in the control group, reported fewer side effects after treatment.

16. In your opinion, what level of evidence does this scenario provide for the effectiveness of Drug X?

| (it provides no evidence)  1 | 2 | 3 | 4 | 5 | 6 | 7 | 8 | 9 | (it provides all the evidence)  10 |
| --- | --- | --- | --- | --- | --- | --- | --- | --- | --- |
| ° | ° | ° | ° | ° | ° | ° | ° | ° | ° |

17. How effective is Drug X?

| (not at all effective)  1 | 2 | 3 | 4 | 5 | 6 | 7 | 8 | 9 | (it is completely effective)  10 |
| --- | --- | --- | --- | --- | --- | --- | --- | --- | --- |
| ° | ° | ° | ° | ° | ° | ° | ° | ° | ° |

**Scenario**

This study aimed to determine how effectively Drug X alleviates the facial pain left behind after infection with the herpes zoster virus.

A total of 250 participants from the Republic of Croatia (130 women and 120 men of all age groups) participated in this study. All participants recovered from herpes zoster infection and had residual pain in the facial area innervated by the nerves affected by the virus.

The participants were randomly divided into two groups (125 respondents each), and neither the participants nor the researchers knew which participant belonged to which group. One group of participants (intervention group) took the painkiller Drug X twice a day after meals for one month, while the other group of subjects (control group) took ibuprofen at a dose of 600 mg twice a day for one month. Daily, the respondents marked the pain level immediately before the treatment and two hours after on a scale from 0 to 10. Also, possible side effects of the treatment were recorded.

The results showed that participants in the intervention group had a lower pain level two hours after treatment than subjects in the control group and had fewer side effects.

18. In your opinion, what level of evidence does this scenario provide for the effectiveness of Drug X?

| (it provides no evidence)  1 | 2 | 3 | 4 | 5 | 6 | 7 | 8 | 9 | (it provides all the evidence)  10 |
| --- | --- | --- | --- | --- | --- | --- | --- | --- | --- |
| ° | ° | ° | ° | ° | ° | ° | ° | ° | ° |

19. How effective is Drug X?

| (not at all effective)  1 | 2 | 3 | 4 | 5 | 6 | 7 | 8 | 9 | (it is completely effective)  10 |
| --- | --- | --- | --- | --- | --- | --- | --- | --- | --- |
| ° | ° | ° | ° | ° | ° | ° | ° | ° | ° |

**Scenario**

In this study, the authors searched the medical research databases in search of all studies that compared the effect of Painkiller X with the effect of a placebo or another painkiller (ibuprofen, paracetamol, tramadol, etc.) in people who had their wisdom tooth surgically removed in the last 7 days.

A literature search resulted in 17 studies that included a total of 560 participants and that compared Drug X with a placebo or another pain reliever (ibuprofen 400 mg or paracetamol 500 mg).

Nine studies compared Drug X with ibuprofen 400 mg (275 participants), five studies compared it with paracetamol 500 mg (181 participants), and three compared it with placebo (104 participants).

An analysis of the results of all studies was done, and the results of nine studies comparing Drug X and ibuprofen 400 mg showed that there is moderate to high-quality evidence that Drug X is more effective than ibuprofen 400 mg in reducing pain after surgical extraction of wisdom teeth.

The results of studies comparing Drug X with paracetamol 500 mg showed that there is high-quality evidence that Drug X is more effective than paracetamol 500 mg in relieving pain after wisdom tooth extraction surgery.

Further, studies comparing Drug X with a placebo showed moderate to high-quality evidence that Drug X is more effective than placebo in relieving pain after wisdom tooth extraction surgery.

20. In your opinion, what level of evidence does this scenario provide for the effectiveness of Drug X?

| (it provides no evidence)  1 | 2 | 3 | 4 | 5 | 6 | 7 | 8 | 9 | (it provides all the evidence)  10 |
| --- | --- | --- | --- | --- | --- | --- | --- | --- | --- |
| ° | ° | ° | ° | ° | ° | ° | ° | ° | ° |

21. How effective is Drug X?

| (not at all effective)  1 | 2 | 3 | 4 | 5 | 6 | 7 | 8 | 9 | (it is completely effective)  10 |
| --- | --- | --- | --- | --- | --- | --- | --- | --- | --- |
| ° | ° | ° | ° | ° | ° | ° | ° | ° | ° |
